# A Physiological Neural Controller of a Muscle Fiber Oculomotor Plant in Horizontal Monkey Saccades

**DOI:** 10.1155/2014/406210

**Published:** 2014-05-07

**Authors:** Alireza Ghahari, John D. Enderle

**Affiliations:** Department of Biomedical Engineering, University of Connecticut, 260 Glenbrook Road, Storrs, CT 06269, USA

## Abstract

A neural network model of biophysical neurons in the midbrain is presented to drive a muscle fiber oculomotor plant during horizontal monkey saccades. Neural circuitry, including omnipause neuron, premotor excitatory and inhibitory burst neurons, long lead burst neuron, tonic neuron, interneuron, abducens nucleus, and oculomotor nucleus, is developed to examine saccade dynamics. The time-optimal control strategy by realization of agonist and antagonist controller models is investigated. In consequence, each agonist muscle fiber is stimulated by an agonist neuron, while an antagonist muscle fiber is unstimulated by a pause and step from the antagonist neuron. It is concluded that the neural network is constrained by a minimum duration of the agonist pulse and that the most dominant factor in determining the saccade magnitude is the number of active neurons for the small saccades. For the large saccades, however, the duration of agonist burst firing significantly affects the control of saccades. The proposed saccadic circuitry establishes a complete model of saccade generation since it not only includes the neural circuits at both the premotor and motor stages of the saccade generator, but also uses a time-optimal controller to yield the desired saccade magnitude.

## 1. Introduction 

Saccades are described as fast eye movements in which a target is tracked by registering the image of that target on the fovea. The saccade neural network requires involvement of a series of neurons designed to imitate the behavior of actual neuronal populations in the horizontal saccade controller. A generic neuron model is therefore desired to quantify the neural stimulations meticulously, thus reflecting the physiology linked to the dendrite, cell body, axon, and presynaptic terminal of each neuron. The continuing research effort in demonstrating such a model has been driven by the need to provide the means to develop a network of neurons, tailored to the complexity involved with inherent physiological evidence. To encompass all of the desired neural behaviors for the other neurons, several modifications to the generic neuron model seem necessary that directly impact its firing rate trajectory [1–3].

The widespread use of spiking neural networks (SNNs) lies in leveraging efficient learning algorithms to the spike response models [4]. A spike pattern association neuron identified five classes of spike patterns associated with networks of 200, 400, and 600 synapses, with success rates of 96%, 94%, and 90%, respectively [5]. Hybrid analog-digital circuitry was laid out to implement an SNN that outputs the postsynaptic potential by integrating the filtered action potentials [6]. Brainstem saccadic circuitry, corroborated by several contributions of local field potentials (LFPs) to the dynamics of neuronal synaptic activity between three neural populations in generating horizontal and vertical saccades in two rhesus monkeys, was introduced by van Horn et al. [7]. The extracellular recordings, including spike trains and LFPs, were taken from the saccadic burst neurons (SBNs) in the paramedian pontine reticular formation (PPRF) at the premotor level, the omnipause neurons in the nucleus raphe interpositus, and the motoneurons at the motor level. It was concluded that LFPs from each neuron encode the eye velocity in both the ipsilateral and contralateral directions. In addition, LFP response amplitude of the SBNs was described as a function of saccade direction (in 400 saccades) by fitting Gaussian curves to data (see Figure 8(B) in [7]), indicating that the SBN LFPs can be fine-tuned over all the directed saccades. A neural system comprised of a persistent firing sensory neuron, a habituating synapse, and a motoneuron was developed to illustrate the spike-timing dependency of the working memory [8]. The persistent firing neuron stems from the Izhikevich neuron model [9], the habituating synapse is a conductance-based model, and the motor neuron captures the essence of the Hodgkin Huxley (HH) model [10]. These studies provide abundant evidence that an SNN is well suited to evoke the properties of the firing patterns of the premotor neurons during the pulse and slide phases of a saccade. However, none of the studies have presented a demonstration of the neural circuits reproducing electrophysiological responses in a network of neurons at both premotor and motor levels. To encompass all of the desired neural behaviors, neural circuitry is used to match the firing rate trajectory of the premotor neurons [3]. We model the saccade-induced spiking activities at the premotor level with an HH model for the bursting neurons and with a modified FitzHugh-Nagumo (FHN) model [11] for the tonic spiking neurons.

Time-optimal control theory of the horizontal saccades establishes the fact that there is a minimum time required for the eyes to reach their destination by involving thousands of neurons. Conjugate goal-directed horizontal saccades were well characterized by a first-order time-optimal neural controller [3]. The analytical solutions of neural innervation signals and the active-state tensions were found to be well matched to the experimental data. It is important that this new, more complex time-optimal controller ascertains that the firing rate of the motoneurons does not change as a function of saccade magnitude during the pulse innervation of the oculomotor plant.

The muscle fiber model (MFM) improves the oculomotor plant model by using several configurations of muscle fibers in series or parallel to drive the eyes to their destination [12]. In other words, it elevates the whole muscle model [1] to the level of muscle fiber model by calculating the viscosity and elasticity of the latter model in terms of the parameter values in the former model. As demonstrated, increasing the number of muscle fibers results in a closer saccadic agreement between the two muscle models [12]. It is indicated that the muscle fiber model substantiates the fact that the number of motoneurons firing has the highest influence on the accuracy of saccade controller, contradicting the control strategy of adjusting the firing rates among the whole neurons. Investigation of muscle fiber model is imperative because it allows for recognizing the effects of the firing of individual neurons, as well as the number of active neurons firing maximally, in controlling the saccades. This investigation as well provides an optimum fit for the agonist and antagonist neural controllers to match the experimental data for the small saccades.

In this paper, we focus on neural control of horizontal monkey saccades. A neural network model of saccade-related neural sites in the midbrain is first presented. We next characterize the underlying dynamics of each neural site in the network, which needs to be treated in the case of spiking neurons. In consequence, to match the dynamics of the neurons and the synapses, saccadic circuitry, including omnipause neuron (OPN), premotor excitatory burst neuron (EBN), inhibitory burst neuron (IBN), long lead burst neuron (LLBN), tonic neuron (TN), interneuron (IN), and motoneurons of abducens nucleus (AN) and oculomotor nucleus (ON), is developed. Finally, the motoneuronal control signals drive a time-optimal controller that stimulates a MFM model of the oculomotor plant. We abbreviate the “conjugate goal-directed horizontal monkey saccade” with the term “saccade” throughout the paper. The terms “motoneurons” and “agonist (antagonist) neurons” are also substitutable in this paper.

## 2. Neural Network

**Figure 1:**
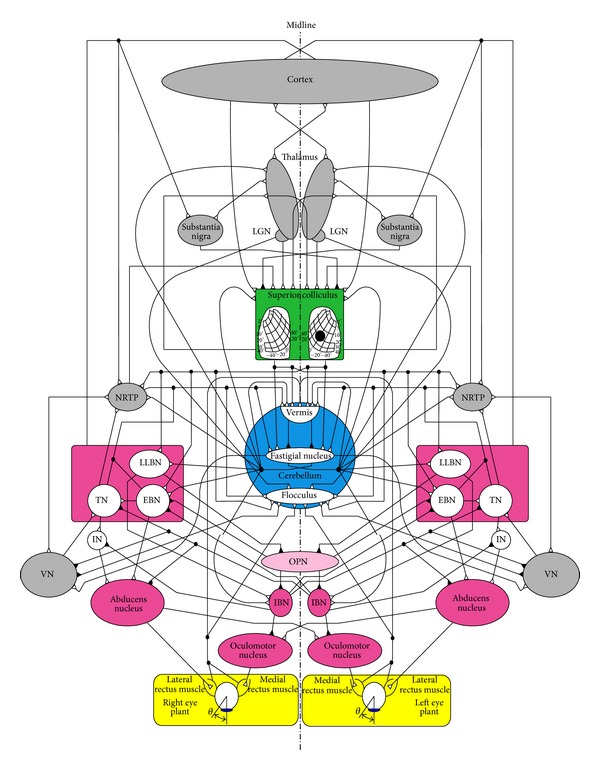
The parallel-distributed neural network for generation of a conjugate goal-directed horizontal saccade in both eyes. Excitatory and inhibitory inputs are shown with white and black triangles at the postsynaptic neurons, respectively. This network is an updated network of that proposed by Enderle and Zhou [3] such that IN mediates between TN and abducens nucleus. In addition, the IN is inhibited by the IBN on each side.

Neurophysiological evidence and developmental studies indicate that important neural populations, consisting of the cerebellum, superior colliculus (SC), thalamus, cortex, and other nuclei in the brainstem, are involved in the initiation and control of saccades [1–3, 13–15]. The studies also provided evidence that saccades are generated through a parallel-distributed neural network, as shown in Figure 1. Neural coordinated activities of the SC and the fastigial nucleus (FN) of the cerebellum are identified as the saccade initiator and terminator, respectively. The two sides of the symmetric network in Figure 1 are known as the ipsilateral side and the contralateral side. The ipsilateral side exhibits coordinated activities in the initiation and control of the saccade in the right eye, while the contralateral side simultaneously synapses with the ipsilateral side to generate a saccade in the left eye. Each neuron in the parallel-distributed network fires in response to other neurons to stimulate the final motoneurons on both sides of the network in a determined manner to execute a saccade. The neural populations on each side of the midline excite and inhibit one another sequentially to ensure that this coactivation leads to the coordination of movement between the eyes.

In the context of the neuroanatomical connectivity structure in Figure 1, the saccade neural network includes neuron populations to imitate the behavior of actual neuronal populations in the initiation, control, and termination of the saccadic burst generator. A description of the synaptic properties of the major neural sites involved in execution of a saccade provides the basis for developing quantitative computational models of the neural network. Here, we outline the characteristics of the premotor neurons in the PPRF and the IN. The synaptic properties of all the other neural sites are explained in [3].

### 2.1. Premotor Neurons in the PPRF

The PPRF encompasses neurons that show dominantly increasing burst frequencies of up to 1,000 Hz during the saccade and remain inactive during the periods of fixation. The LLBN and the medium lead burst neuron (MLBN) are the two types of burst neurons in the PPRF. The LLBN forms an excitatory synapse to the IBN and an inhibitory synapse to the OPN.

There are two types of neurons in the MLBN: the EBN and the IBN. The EBN serves as one of the vital excitatory inputs for the saccade controller. The primary inputs to this neuron are the excitatory input of the SC and the inhibitory input from the contralateral IBN and OPN. This neuron forms excitatory synapses to the TN and the AN. The IBN, though, controls the firing of the EBN as well as the TN, both of which are on the opposite side of the network to the corresponding IBN. It also inhibits the ON and the IN on the same side as itself. This neuron receives excitatory inputs from the FN of the cerebellum on the opposite side and the LLBN on the same side and an inhibitory input from the OPN.

### 2.2. Interneuron (IN)

Many excitatory and inhibitory INs in the central nervous system stimulate and control motoneurons. The cerebellum aggregates most of these INs whose functionality depends on the anatomical aspects and properties of their membranes. The IN receives the excitatory and inhibitory inputs from the corresponding TN and IBN, respectively. It consecutively provides the step component to the agonist and antagonist neural controllers. As with the TN, the utility of the modified FHN model under the tonic bursting mode exhibits this particular neural spiking activity [11]. The following section characterizes the underlying dynamics of each neural site in the neural network.

## 3. Firing Characteristics of Each Type of Neuron

The saccade generator investigated in this work is built upon the extant research [1–3, 13, 14, 16, 17]. Monkey saccades are categorized into two different modes of operation, namely, small (ranging from 3° to 8°) and large (above 8°) [3]. The differentiation between these two modes has been governed by the fact that when the saccade size increases, more active neurons are firing synchronously to form the agonist neural input for small saccades. For large saccades, however, the number of active neurons firing maximally remains unchanged, consistent with the time-optimal controller described by Enderle and Wolfe [18]. The model is first-order time-optimal; that is, it does not depend on the firing rate of the neurons to determine the saccade magnitude. We next demonstrate features of the structure of the proposed saccade neural network to highlight the important neurological control implications.

### 3.1. Neural Activity

The structure of the saccade neural network leverages neural coding so that burst duration is transformed into saccade amplitude under the time-optimal condition. Such coding manifests activities, including the onset of burst firing before saccade, peak firing rate, and end of firing with respect to the saccade termination, for each neuron on the basis of the physiological evidence. These characteristics are provided for the neural sites [3] as a framework for our simulations.  Table 1 summarizes the activities in initiating, controlling, and terminating the burst firing through the neural network, generating a saccade in the right eye. Note that the agonist and antagonist tonic firing is governed by the ipsilateral IN activity under the tonic firing operation mode [11].

### 3.2. Burst Discharge Mechanism

**Figure 2:**
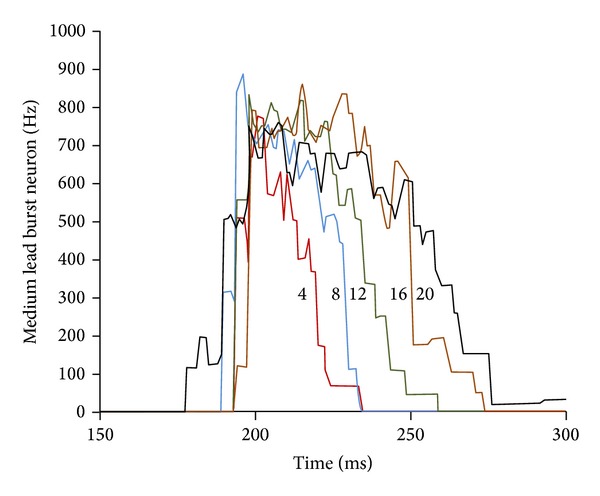
The firing rate trajectories for a medium lead burst neuron for saccades of 4°, 8°, 12°, 16°, and 20° [12].

The firing rate trajectories of a medium lead burst neuron of monkey data for saccades of 4°, 8°, 12°, 16°, and 20° are provided [12]. It is explained that such trajectories are in agreement with the data published in the literature [19, 20]. This illustration of the trajectories in Figure 2 here aids in comprehending the foundations of the first-order time-optimal neural controller. The entire active agonist neurons fire maximally during the pulse interval of the saccade. For small saccades, the controller is constrained by a required minimum duration of the agonist pulse. Knowing this, the saccade magnitude depends on the number of active neurons, firing maximally, in accord with the physiological evidence [3, 12]. Note that the number of active neurons is the only parameter that varies in the MFM among different saccades in an adaptive control strategy of the oculomotor plant. It is demonstrated [3, 12] that adjusting this parameter provides significant analytical convenience in controlling the small saccades as opposed to changing the firing rate of all active neurons as a function of saccade magnitude. For the large saccades, the duration of the agonist pulse is the dominant factor that determines the saccade magnitude according to the main-sequence diagrams [3]. Such duration varies noticeably among the large saccades shown in Figure 2. The FN in the cerebellum records the duration of the agonist pulse and the number of active neurons in arranging the end of the saccade.

As motoneurons receive excitatory input from the ipsilateral EBN, the burst discharge in them during a saccade is adequately similar to the EBN bursting. Such burst discharge in the motoneurons is responsible for the movement of the rectus muscles during a saccade. The firing rate trajectory of the EBN is of prime importance in control of such a saccade. The presented EBN model [3] showed a constant plateau of bursting during the second portion of the burst before the decay occurs [18]. We model the EBN firing rate by applying the firing rate trajectory in which a slow linear reduction in firing rate is assumed [3, 21]. We also consider this trajectory for the SC current stimulation of the LLBN, which is in accord with the different simulations in examining the effects of several depolarizing stimulus currents in the EBN axon (specifically, see Figure 2.10 in [3]). It should be emphasized at this point how the SC contributes to the optimal control of the saccades by driving the LLBN. The movement fields within the SC are indicators of the number of neurons firing for different small and large saccades (see locus of points on a detailed view of the SC retinotopic mapping in Figure 2.14 in [3]). It is implied that the number of cells firing in the LLBN is determined by the number of cells firing in the SC as long as there is a feedback error maintained by the cerebellar vermis [3]. The number of the OPN cells firing after inhibition from the LLBN determines, in turn, how many EBN cells are released from inhibition. Finally, the number of EBN cells firing determines the number of motoneurons driving the eyes to their destination.

### 3.3. Sequence of Neural Firing

**Figure 3:**
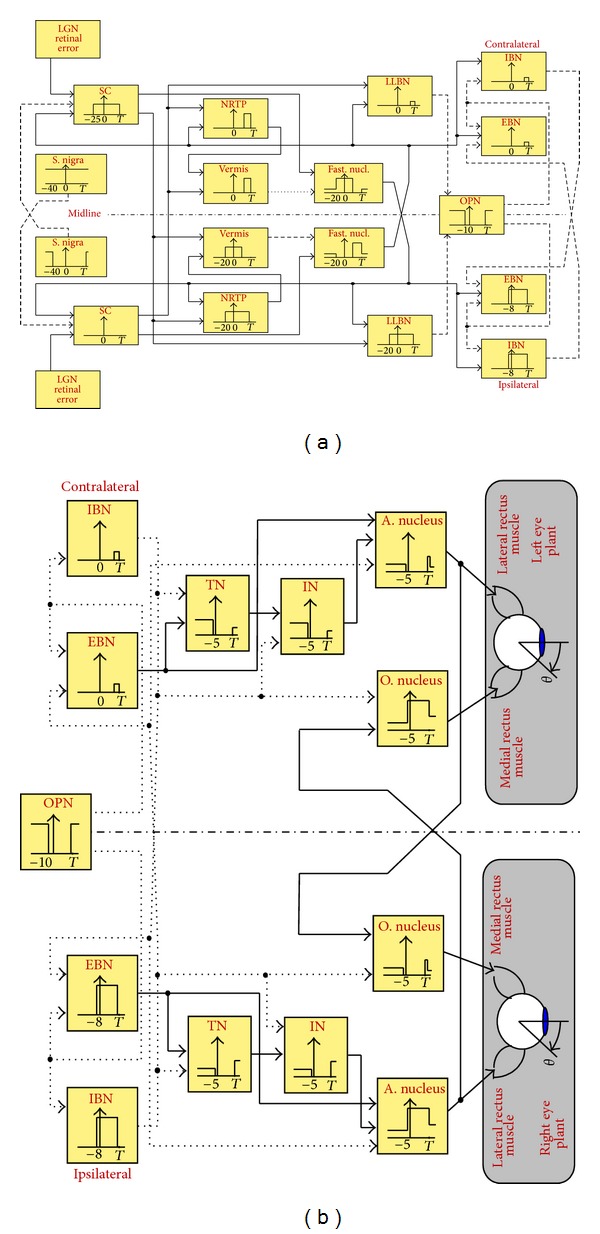
A functional block diagram of the saccade generator model [3]. Solid lines are excitatory and dashed lines are inhibitory. Each block represents the neural activity at each neural site as indicated in Table 1. (a) Neural pathways from the formation of the lateral geniculate nucleus (LGN) retinal error to the MLBN activity. (b) Neural pathways from the MLBN to the rectus muscles in both eyes.

The saccade completion involves the evolution of some events in an orderly sequence in the neural sites. Such neural sites are shown in Figure 3 via a functional block diagram [3]. The output of each block indicates the firing pattern at each neural site manifested during the saccade: saccade starts at time zero, and *T* represents the saccade termination. The negative time for each neural site refers to the onset of the burst before saccade (see Table 1). The neural activity within each block is represented as pulses and/or steps, consistent with the described burst discharge mechanism, to reflect the neural operation as timing gates [3]. Ultimately, motoneurons innervate rectus muscles in both eyes at the end interaction level of the block diagram.

The following description outlines eight steps required to implement the saccade control strategy in the context of Figure 3. It represents the sequence of events accounted for by Enderle and Zhou [3], with modifications made in steps (iv)–(vii) to indicate the function of local neural integrators (TN and IN) in providing the step component to the motoneurons.

- The deep layers of the SC initiate a saccade based on the distance between the current position of the eye and the desired target.
- The ipsilateral LLBN and EBN are stimulated by the contralateral SC burst cells. The LLBN then inhibits the tonic firing of the OPN. The contralateral FN also stimulates the ipsilateral LLBN and EBN.
- When the OPN ceases firing, the MLBN (EBN and IBN) is released from inhibition.
- The ipsilateral IBN is stimulated by the ipsilateral LLBN and the contralateral FN of the cerebellum. When released from inhibition, the ipsilateral EBN responds with a postinhibitory rebound burst for a brief period of time. The EBN, when stimulated by the contralateral FN (and perhaps the SC), enables a special membrane property that causes a high-frequency burst that decays slowly until being inhibited by the contralateral IBN. The burst firing activity of EBN is integrated through the connection with the TN. The IN follows closely the same integration mechanism as that of the TN.
- The burst firing in the ipsilateral IBN inhibits the contralateral EBN, IN, and AN, as well as the ipsilateral ON.
- The burst firing in the ipsilateral EBN causes the burst in the ipsilateral AN, which then stimulates the ipsilateral lateral rectus muscle and the contralateral ON. With the stimulation of the lateral rectus muscle by the ipsilateral AN and the inhibition of the ipsilateral medial rectus muscle via the ON, a saccade occurs in the right eye. Simultaneously, the contralateral medial rectus muscle is stimulated by the contralateral ON, and, with the inhibition of the contralateral lateral rectus muscle via the AN, a saccade occurs in the left eye. Hence, the eyes move conjugately under the control of a single drive center. During the fixation periods, the INs provide the steady-state tensions required to keep the eyes at the desired destination.
- At the termination time, the cerebellar vermis, operating through the Purkinje cells, inhibits the contralateral FN and stimulates the ipsilateral FN. Some of the stimulation of the ipsilateral LLBN and IBN is lost because of the inhibition of the contralateral FN. The ipsilateral FN stimulates the contralateral LLBN, EBN, and IBN. The contralateral EBN then stimulates the contralateral AN. The contralateral IBN then inhibits the ipsilateral EBN, TN, and AN and contralateral ON. This inhibition removes the stimulus to the agonist muscle.
- The ipsilateral FN stimulation of the contralateral EBN allows for modest bursting in the contralateral EBN. This activity then stimulates the contralateral AN and ipsilateral ON. Once the SC ceases firing, the stimulus to the LLBN stops, allowing the resumption of OPN firing that inhibits the ipsilateral and contralateral MLBN, hence terminating the saccade.

The advances in computational neural modeling have supplied us with abundant information at different structural scales, such as the biophysical [4, 5], the circuit [3, 6, 7], and the systems levels [8]. The following includes our modeling of the premotor and motor neurons at the circuit level. We introduce a neural circuit model that can be parameterized to match the described firing characteristics of each type of neuron.

## 4. Neural Modeling

**Figure 4:**
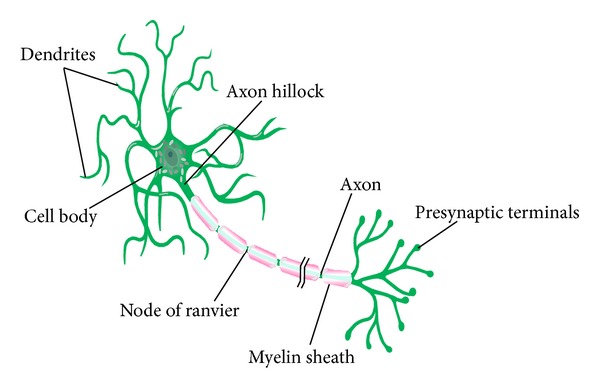
A schematic presentation of the different components of a neuron [16].

A typical neuron embodies four major components: cell body, dendrites, axon, and presynaptic terminals, as shown in Figure 4. The neural cell body encompasses the nucleus and is similar to the other cells. Dendrites act as the synaptic inputs for the preceding excitatory and inhibitory neurons. Upon this stimulation of the neuron at its dendrites, the permeability of the cell's plasma membrane to sodium intensifies, and an action potential moves from the dendrite to the axon [16]. The transmission of action potential along the axon is facilitated by means of nodes of Ranvier in the myelin sheath. At the end of each axon, there are presynaptic terminals, from which the neurotransmitters diffuse across the synaptic cleft.

A complete understanding of the properties of a membrane by means of standard biophysics, biochemistry, and electronic models of the neuron will lead to a better analysis of membrane potential response. This response is dependent on how much neurotransmitter is received from the presynaptic terminal of the adjacent neurons; thereby, the neuron becomes hyperbolized or depolarized. A generic neuron circuit model is introduced in this section, together with the description of the modifications required to populate a neural network for control of saccades. The saccade neural network includes eight neuron populations at premotor and motor levels as seen in Figure 1:

- long lead burst neuron (LLBN),
- omnipause neuron (OPN),
- excitatory burst neuron (EBN),
- inhibitory burst neuron (IBN),
- tonic neuron (TN),
- interneuron (IN),
- abducens nucleus (AN),
- oculomotor nucleus (ON).

The saccade circuitry underlies the dynamics of the above eight distinct neurons, each of which contributes to the control mechanism of the saccade. Except for the OPN, the proposed parallel-distributed neural network proposed parallel-distributed neural network accommodates two of each of the other neurons in the network. The dendrite model delineated below is adjustable to the stimulation mechanism of all eight neurons. The axon model for all spiking neurons, except the EBN and OPN, adheres to the Hodgkin-Huxley (HH) model. The EBN and OPN are neurons that fire automatically when released from inhibition—these neurons are modeled using a modified HH model [3]. The TN integrates its input and is modeled with a FitzHugh-Nagumo (FHN) model under the tonic bursting mode [11]. The presynaptic terminal elicits a pulse train stimulus whose amplitude depends on the membrane characteristics of the postsynaptic neuron.

### 4.1. Dendrite Model

**Figure 5:**
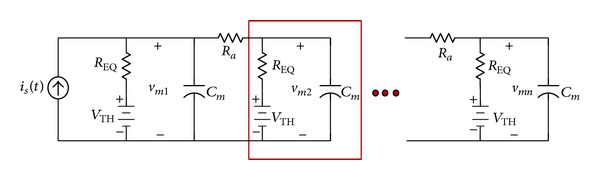
The dendrite circuit model with *n* passive compartments: *i*_*s*_(*t*)models the stimulus current from the adjacent neurons to the dendrite. Each compartment has membrane electromotive, resistive, and capacitive properties—*V*_TH_, *R*_EQ_, and *C*_*m*_ in the second compartment are noted. The batteries in the circuit, *V*_TH_, are the Thevenin equivalent potential of all the ion channels. The axial resistance *R*_*a*_ connects each compartment to the adjacent ones (remains unchanged among the neurons). Appropriate values for the membrane resistance and capacitance of the dendrite model are found to match the firing characteristics of each type of neuron.

The dendrite is partitioned into a number of membrane compartments, each of which has predetermined length and diameter. Each compartment in the dendrite has three passive electrical characteristics: electromotive force (emf), resistance, and capacitance, as shown in Figure 5. Axial resistance is used to connect the dendrite to the axon.

The presynaptic input to the dendrite is modeled as a pulse train current source (*i*_*s*_). The node equation for the first dendrite compartment is

(1) $$\begin{array}{c} \;C_{m}\frac{dv_{m1}}{dt}+\frac{v_{m1}-V_{\text{TH}}}{R_{\text{EQ}}}+\frac{v_{m1}-v_{m2}}{R_{a}}=i_{s}, \end{array}$$

where *v*_*m*1_ is the membrane potential of the first compartment and *v*_*m*2_ is the membrane potential of the second compartment. The membrane resistance *R*_EQ_, capacitance *C*_*m*_, and the emf *V*_TH_ characterize each compartment. *R*_*a*_ is the axial resistance.

For all intermediate dendrite compartments, there are two inputs: the input from the previous compartment's membrane potential and the input from the next compartment's membrane potential. The node equation for the second compartment is

(2) $$\begin{array}{c} C_{m}\frac{dv_{m2}}{dt}+\frac{v_{m2}-V_{\text{TH}}}{R_{\text{EQ}}}+\frac{v_{m2}-v_{m1}}{R_{a}}+\frac{v_{m2}-v_{m3}}{R_{a}}=0, \end{array}$$

where *v*_*m*3_ is the membrane potential of the third compartment.

The last dendrite compartment receives just one input from its preceding compartment. The corresponding node equation is

(3) $$\begin{array}{c} \;C_{m}\frac{dv_{mn}}{dt}+\frac{v_{mn}-V_{\text{TH}}}{R_{\text{EQ}}}+\frac{v_{mn}-v_{m(n-1)}}{R_{a}}=0, \end{array}$$

where the membrane potential *v*_*mn*_ is related to the preceding compartment's membrane potential (*v*_*m*(*n*−1)_) through the axial resistance *R*_*a*_.

The neurons' dendrite model is realized by experimental tuning of the parametric capacitance and resistance properties of the basic dendrite model. This parametric adaptation allows for the accommodation of the synaptic transmission in the neural network as required to stimulate each postsynaptic neuron. Each neuron's dendrite rise time constant determines the delay to emulate the postsynaptic potential propagation along the dendrite, consistent with the initiation of firing with respect to the saccade onset provided in Table 1.  Table 2 includes the membrane resistance and capacitance of the dendrite compartments for each neuron.

**Figure 6:**
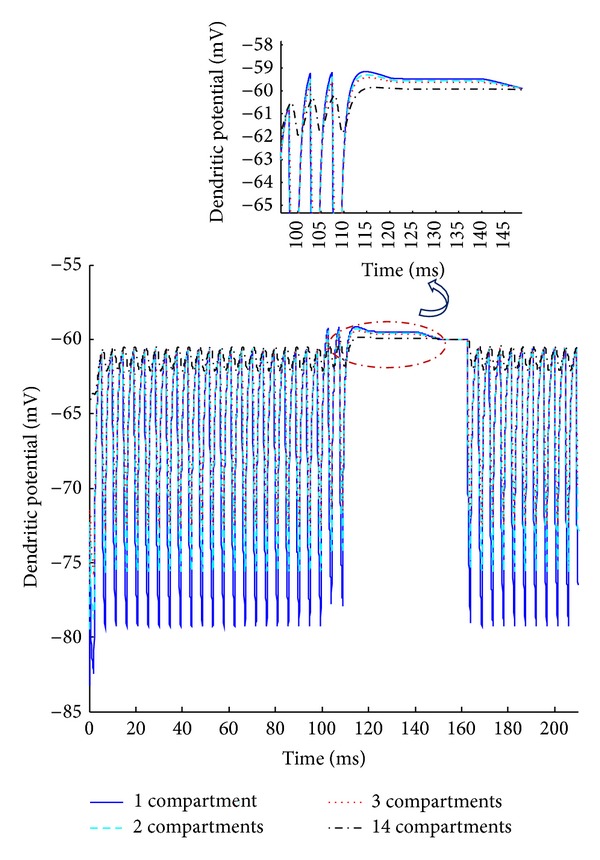
The EBN dendritic membrane potential across the different compartments. The corresponding interval of burst firing is emphasized. The membrane parameter values are *V*_TH_ = −60 mV, *C*_*m*_ = 0.45 *μ*F, *R*_EQ_ = 3.1 kΩ, and *R*_*a*_ = 100 Ω.

Initial condition of the capacitor is set to *V*_TH_ at steady state. Computational efficiency accrues when the minimum number of compartments in the dendrite model is required. We chose to include 14 compartments in the dendrite to achieve the desired membrane properties in each type of neuron. For example, the EBN dendritic membrane potential across the first, second, third, and last compartments is illustrated in Figure 6. The farther the compartment is along the dendrite, the smoother its potential response is to the pulse train current source.

### 4.2. Axon Model

**Figure 7:**
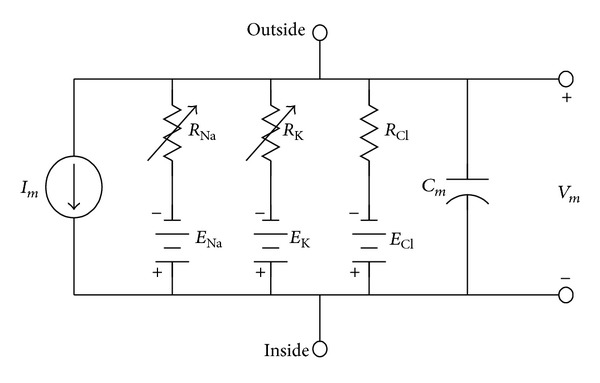
The circuit model of an unmyelinated portion of squid giant axon [3]. The variable active gate resistances for Na^+^ and K^+^ are given by $R_{\text{K}}=1/{\overset{-}{g}}_{\text{K}}N^{4}$ and $R_{\text{Na}}=1/{\overset{-}{g}}_{\text{Na}}M^{3}H$, respectively. The passive gates are modeled by a leakage channel with resistance, *R*_*l*_ = 3.33 kΩ. The battery is the Nernst potential for each ion: *E*_*l*_ = 49.4 V, *E*_Na_ = 55 V, and *E*_K_ = 72 V.

The Hodgkin-Huxley (HH) model of the axon serves as the basis for the neurons modeled here—only the EBN and OPN are based on a modified HH model [3]. This nonlinear model describes the membrane potential at the axon hillock caused by conductance changes. The circuit diagram of an unmyelinated portion of squid giant axon is illustrated in Figure 7. The node equation that expresses the membrane potential *V*_*m*_ as a function of stimulus current *I*_*m*_ from the dendrite and voltage-dependent conductance of the sodium and potassium channels is [3]

(4) g−KN4(Vm−Ek)+g−NaM3H(Vm−ENa)  +(Vm−El)Rl+CmdVmdt=Im,

where

(5) $$\begin{array}{c} \frac{dN}{dt}=\alpha_{N}\left(1-N\right)-\beta_{N}N,\\ \frac{dM}{dt}=\alpha_{M}\left(1-M\right)-\beta_{M}M,\\ \frac{dH}{dt}=\alpha_{H}\left(1-H\right)-\beta_{H}H,\\ {\overset{-}{g}}_{\text{K}}=36\times 10^{-3}\;\text{S},\;\;{\overset{-}{g}}_{\text{Na}}=120\times 10^{-3}\;\text{S}. \end{array}$$

The coefficients in the above first-order system of differential equations are related exponentially to the membrane potential *V*_*m*_; that is,

(6) αN=0.01×V+10e((V+10)/10)−1 ms−1,βN=0.125e(V/80) ms−1,αM=0.1×V+25e((V+25)/10)−1 ms−1,βM=4e(V/18) ms−1,  αH=0.07e(V/20) ms−1,βH=1e((V+30)/10)+1 ms−1,V=Vrp−Vm mV,

where the resting potential *V*_rp_ is −60 mV.

The neural firing rate of the entire burst neurons has been adjusted to meet the peak firing rate requirement in Table 1. This adjustment is intended for each neuron to contribute to the generation of the saccade by mimicking the required physiological properties [3]. To this end, the right-hand side of the *N*, *M*, and *H* differential expressions in (4) is multiplied by appropriate coefficients to achieve the desired peak firing rates. For instance, the required coefficient for the EBN has been 35,000; thereby, it presents a peak firing rate at 1,000 Hz. Note that the above description of the basic HH model of the axon has been used for all burst neurons, except for the EBN and the OPN. For these latter neurons, the modified HH model is used to change the threshold voltage from −45 mV to −60 mV. Through illustrative examples, it has been shown that this variation causes EBN to fire autonomously without the existence of any excitatory stimulus [3]. From the description of the dominant effect of the sodium channel current on the changes in threshold voltage at the beginning of the action potential [3], threshold voltage in the EBN axon model is changed by modifying the *α*_*M*_ equation to

(7) $$\begin{array}{c} \alpha_{M}=0.1\times \frac{V+10}{e^{\left(\left(V+10\right)/10\right)}-1}\;\text{m}{\text{s}}^{-1}. \end{array}$$

The OPN axonal threshold voltage of firing has been adjusted following the same modification by (7). This alteration of the threshold voltage for the EBN and the OPN enables them to fire spontaneously without any significant depolarization from peripheral current stimuli.  Table 2 lists the firing threshold voltage and the coefficient required to adjust the peak firing rate for each neuron.

The axon transfers an action potential from the spike generator locus to the output end of the synaptic mechanism. The transmission along the axon thus amounts to introducing a time delay, after which the action potential appears at the synapse.

### 4.3. Synapse Model

**Figure 8:**
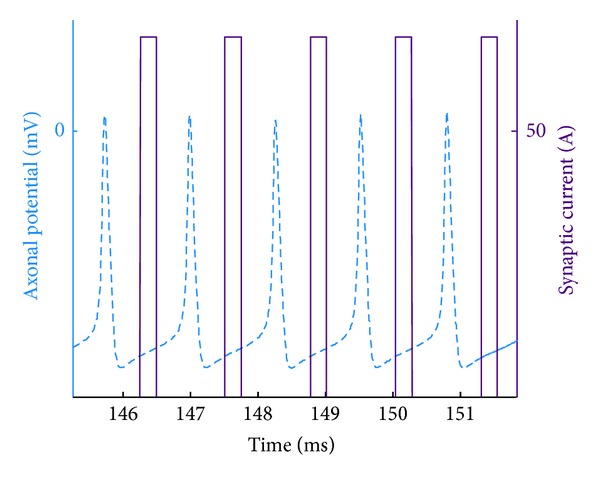
A train of action potentials (dashed) and current pulses (solid) reflecting the synaptic mechanism of the EBN. Each current pulse shows a time delay with respect to the corresponding action potential, attributable to the transmission delay along the axon.

When the action potential appears at the synapse, packets of neurotransmitters are released. This is modeled by excitatory or inhibitory pulse train stimuli to stimulate the dendrite of the postsynaptic neuron more realistically. In the chain of synaptic transmission, the amplitude and width of each single pulse are chosen experimentally to provide the desired postsynaptic behavior in the neurons based on timing constraints in Table 1. The width is constrained by the two points at which the action potential crosses a constant level of the axonal potential. The synapse can be in that sense thought of as a voltage-to-frequency converter that releases a pulse train output.  Figure 8 shows a number of action potentials and the synaptic current pulses of the EBN toward the end of the burst firing interval. Note that the time delay between each action potential and the corresponding current pulse is evident.

In addition to the transmission time delay along the axon, all chemical synapses introduce a small delay before excitatory or inhibitory pulse train. This delay accounts for the time required for the release of neurotransmitters and the time it takes for them to distribute through the synaptic cleft. This small synaptic delay was taken into effect by increasing the rise time constant of the following postsynaptic dendritic compartments.

As indicated, the amplitude and width of synaptic current pulses for each neuron are uniquely chosen in order that the postsynaptic neurons exhibit the desired behavior.  Table 2 includes such amplitude of the synaptic current pulses. This table summarizes all the differences (dendritic, axonal, and synaptic) among eight distinct neurons whose realization is important in the time-optimal control of the saccade.

We next describe a linear homeomorphic muscle model that captures the nonlinear properties of the muscle, namely, force-velocity and length-tension relationships.

## 5. Linear Homeomorphic Model of the Muscle

The time-optimal controller model was investigated to obtain the saccadic eye movement model solution that drives the eyeball to its destination for different saccades [3, 17]. Here, we explain that the saccadic eye movement model solution is characterized by realization of the agonist and antagonist controller models, thereby providing the active-state tensions as inputs to a linear homeomorphic model of the oculomotor plant.

### 5.1. Muscle Neural Stimulation

The first-order time-optimal controller model is defined by two complementary controllers: agonist controller model and antagonist controller model. These models describe how the neural innervation signals from motoneurons are converted to the active-state tensions to drive the agonist and antagonist muscle during the saccade (assuming that there is an oculomotor plant with a given order). In what follows, the active-state tensions are defined as the low-pass filtered neural innervation signals.

#### 5.1.1. Agonist Controller Model

The agonist controller is a first-order pulse-slide-step neuronal controller that describes the agonist active-state tension as the low-pass filtered neural stimulation signal [3]. The neural stimulation signal is the firing rate of the ipsilateral AN and that of the contralateral ON. The slide is meant to model the transition between the pulse and the step exponentially. The expression of low-pass filtering of the neural innervation input to the agonist controller model is

(8) $$\begin{array}{c} {\dot{F}}_{\text{ag}}=\frac{N_{\text{ag}}-F_{\text{ag}}}{\tau_{\text{ag}}}, \end{array}$$

where

(9) $$\begin{array}{c} \tau_{\text{ag}}=\tau_{\text{gac}}\left(u\left(t-t_{1}\right)-u\left(t-t_{2}\right)\right)+\tau_{\text{gde}}u\left(t-t_{2}\right), \end{array}$$

where *N*_ag_ represents the agonist neural innervation input from which the agonist active-state tension, *F*_ag_, is generated. The agonist time constant *τ*_ag_ is expressed by two step functions dependent on the agonist activation time constant, *τ*_gac_, and the deactivation time constant *τ*_gde_. *t*_1_ indicates that the saccade has the latent period, and *t*_2_ is the start of the transition slide interval for the agonist controller. It is noteworthy that the activation (deactivation) time constant in the model accounts for the different dynamic characteristics of muscle upon increasing (decreasing) stimulation.

#### 5.1.2. Antagonist Controller Model

The antagonist muscle is unstimulated by a pause during the saccade and remains fixed by a step input to keep the eyeball at its destination. To serve this purpose, a first-order pause-step neuronal controller is defined [3]. The neural stimulation signal to the controller is the firing rate of the ipsilateral ON and that of the contralateral AN. The antagonist active-state tension can be expressed as the low-pass filtered pause-step waveform:

(10) $$\begin{array}{c} {\dot{F}}_{\text{ant}}=\frac{N_{\text{ant}}-F_{\text{ant}}}{\tau_{\text{ant}}}, \end{array}$$

where

(11) $$\begin{array}{c} \tau_{\text{ant}}=\tau_{\text{tde}}\left(u\left(t-t_{1}\right)-u\left(t-t_{3}\right)\right)+\tau_{\text{tac}}u\left(t-t_{3}\right), \end{array}$$

where *N*_ant_ denotes the antagonist neural innervation input and the *F*_ant_ is the antagonist active-state tension generated. The antagonist time constant is describable by two step functions, introducing the antagonist deactivation time constant, *τ*_tde_, and the activation time constant *τ*_tac_. *t*_1_ takes into account the latent period, and *t*_3_ is the onset of the change to the step component necessary to keep the eyeball steady at its destination.

The time-optimal controller has been found to be reasonably consistent with the characteristics of the main-sequence diagrams [3]. In what follows, a linear homeomorphic muscle fiber model (MFM) of the oculomotor plant is presented.

### 5.2. Oculomotor Plant

A linear homeomorphic MFM that captures the nonlinear properties of the muscle, namely, force-velocity and length-tension relationships, is investigated [12]. The muscle fiber is known as the basic structural unit of the muscle that exhibits the same mechanical functionality as the whole muscle model [3]. The significance of introducing a muscle fiber model is that it accommodates multiple neurons to drive the eyes to their destination. Accordingly, the effect of the number of active neurons in controlling the saccade magnitude can be investigated in an adaptive control paradigm of the oculomotor plant. This muscle neural stimulation control has remarkably suited the investigation of the oculomotor plant [22]. In contrast to the whole muscle model, information about the muscle fibers is not aggregated into just a few parameters in the MFM. The entire 100 muscle fibers are similar in terms of the parameters and the stimulation from the active state tension generator model [12].

The rigorous analysis of the MFM including both the length-tension and the force-velocity characteristics indicates that this model agrees with the previous results from the whole muscle model [3, 12]. The experiments with the MFM are illustrated for different combinations of columns of series of muscle fibers, load mass, and active state tension.

**Figure 9:**
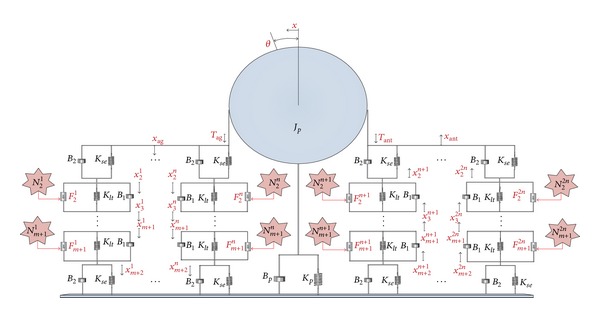
Muscle fiber oculomotor plant for the agonist and antagonist rectus eye muscles [12]. The muscles are assumed to be stretched by 3.705 mm at the primary position.

The focus of our attention herein is the description of an oculomotor plant in which the MFMs of the agonist and antagonist rectus eye muscles are incorporated [12]. The MFM is parameterized using the scaled estimates of the parameters from the whole muscle oculomotor plant [12].  Figure 9 shows the studied oculomotor plant with two parallel networks of the muscle fibers attached to the eyeball. *θ* is the angle of displacement of the eyeball from the primary position, and *x* denotes the respective change in length of arc. The changes in the length of the agonist muscle on the left and the antagonist muscle on the right are shown by *x*_ag_ and *x*_ant_, respectively. There are 2*n* columns of muscle fibers, each of which has *m* muscle fibers in series. Each column of muscle fibers also includes two tendon elements, whose viscous and elastic elements are *B*_2_ and *K*_se_, at the top and bottom of it. Each structural unit of muscle fiber is modeled by a viscous element, *B*_1_, an elastic element, *K*_lt_, and an active state generator, *F*_*j*_^*i*^, where 1 ≤ *i* ≤ 2*n* and 2 ≤ *j* ≤ *m* + 1. The change from the primary position at node *j* in the muscle fiber column *i* is denoted by *x*_*j*_^*i*^. Note that *N*_*j*_^*i*^ exhibits the input to the agonist and antagonist controller models stated formerly. Motoneurons provide the saccadic neural innervation signals to each muscle fiber in our time-optimal controller. The advantage of the state-variables approach facilitated the mathematical descriptions of the oculomotor plant and its implementation in the MATLAB/Simulink [12]. With definition of *y*_1_^*i*^ = *x*_ag_ − *x*_2_^*i*^, the net torque developed by the agonist MFM is

(12) $$\begin{array}{c} T_{\text{ag}}=-\overset{n}{\underset{i=1}{\sum }}\left(K_{\text{se}}y_{1}^{i}+B_{2}{\dot{y}}_{1}^{i}\right), \end{array}$$

where, for the two tendon elements in each column (*j* = 1 and *m* + 2), the state equation is

(13) $$\begin{array}{c} {\dot{y}}_{j}^{i}=\frac{-\left(T_{i}+K_{\text{se}}y_{j}^{i}\right)}{B_{2}}, \end{array}$$

and the state equation that represents the dynamics of muscle fibers in each column (2 ≤ *j* ≤ *m* + 1) is

(14) $$\begin{array}{c} {\dot{y}}_{j}^{i}=\frac{-T_{i}-K_{\text{lt}}y_{j}^{i}+F_{j}^{i}}{B_{1}}, \end{array}$$

where *T*_*i*_ is the tension generated by each muscle fiber column.

In a similar approach for the antagonist MFM (*n* + 1 ≤ *i* ≤ 2*n*), after definition of *y*_1_^*i*^ = *x*_ant_ − *x*_2_^*i*^, the net torque developed by the antagonist MFM is

(15) $$\begin{array}{c} T_{\text{ant}}=\overset{2n}{\underset{i=n+1}{\sum }}\left(K_{\text{se}}y_{1}^{i}+B_{2}{\dot{y}}_{1}^{i}\right), \end{array}$$

where the state equation for the two tendon elements in each column (*j* = 1 and *m* + 2) is

(16) $$\begin{array}{c} {\dot{y}}_{j}^{i}=\frac{T_{i}-K_{\text{se}}y_{j}^{i}}{B_{2}}, \end{array}$$

and the dynamics of muscle fibers in each column (2 ≤ *j* ≤ *m* + 1) are represented by

(17) $$\begin{array}{c} {\dot{y}}_{j}^{i}=\frac{T_{i}-K_{\text{lt}}y_{j}^{i}-F_{j}^{i}}{B_{1}}, \end{array}$$

where *T*_*i*_ denotes the tension developed by each muscle fiber column. Consequently, the third-order linear differential equation to solve for the optimal solution for a saccade is [12]

(18) $$\begin{array}{c} T_{\text{ag}}-T_{\text{ant}}=\frac{J_{p}}{r}\ddot{\theta }+\frac{B_{p}}{r}\dot{\theta }+\frac{K_{p}}{r}\theta , \end{array}$$

where *J*_*p*_ denotes the moment of inertia of the eyeball, *B*_*p*_ denotes the viscous element of the eyeball, and the passive elasticity of the eyeball is represented by *K*_*p*_. *r* is the radius of eyeball (10 mm for monkey). It is assumed that the muscles are primarily stretched by 3.705 mm [1, 3].

Note that the above expressions show that the inputs to the MFM are the agonist and antagonist active-state tensions. These tensions are obtained by low-pass filtering of the motoneurons' innervation signals as previously described for both the pulse-slide-step and pause-step controllers.

The analytical solutions for all *F*_*j*_^*i*^ were yielded in the previous work [12], and it was found that different characteristics of saccades are very well matched to those of the experimental data. The estimation routine [3] involved estimation of 25 parameters of the oculomotor plant, neural inputs, and active-state tensions. The parameters' physiological accuracy was corroborated by the previously published experimental findings for human and monkey. Note that no empirical parameters are involved herein other than the parameters of the whole muscle model of the oculomotor plant for monkey (see page 47 in [3]).

## 6. Simulation Results

Two small saccades (4° and 8°) and three large saccades (12°, 16°, and 20°) have been the focal point of our simulations of horizontal monkey saccades under the first-order time-optimal control strategy. All neural populations consisted of 14 dendrite compartments with membrane properties included in Table 2. The determination of the rise time constant for each neuron's dendrite plays a vital role in the integration of current pulses at the synapse. Analyses of the dendritic membrane potentials were performed with the NI Multisim circuit design suite, and the neural network was simulated in the MATLAB/Simulink environment. The saccade-induced spiking activities at the premotor level are modeled with an HH model for the bursting neurons [3]. The tonic spiking behavior of the TN/IN is implemented by a modified FHN model as well [11]. Transmission along the axon introduced a delay after the presence of action potential at the axon hillock, after which an action potential appears at the synapse. Synaptic connections between functionally modeled neuron populations are modeled following a current-based synapse scheme (see Table 2 for differences in the membrane parameters among the neurons). The onset delay before saccade, peak firing rate, and burst termination time for the different neuron populations are chosen according to Table 1.

We arranged 100 identical muscle fibers (*n* = 1 and *m* = 100), since this coordination provided sufficient resolution in matching the experimental data [12]. As described, the number of active neurons impacts the control of saccades instead of the variations in the firing rate of those neurons under the time-optimal control strategy. In addition, the number of active neurons differs from saccade to saccade, as evident by the dynamics observed in the main-sequence diagrams [3, 12]. As demonstrated, this system parameter is determined by reducing from a maximum of 100 active neurons until the eye position estimate from the MFM and the whole muscle model match [12]. The active-state tension for each of the agonist neurons that are not activated is modeled to exponentially decay (during the pulse) and rise (during the slide) using the same time constants in the agonist controller model.

The number of active agonist neurons for the 4° and 8° saccades is reported to be 48 and 76, respectively [12]. The neural innervations from this number of neurons for each small saccade of the muscle fiber oculomotor plant tend to be in excellent agreement with those of the whole muscle oculomotor plant. Each active neuron exhibits the pause-slide-step firing trajectory as later shown in Figure 11, substantiating the physiological accuracy [23] of the agonist controller model. The adjustment of the number of active neurons for the large saccades is empirically carried out to maximize the correlation between the whole muscle oculomotor plant and the muscle fiber oculomotor plant [12]. The number of active neurons is estimated to be 75 neurons for the 12° saccade, 100 neurons for the 16° saccade, and 92 neurons for the 20° saccade.  Table 3 lists the number of active neurons and the duration of the burst (agonist pulse), for the five different saccades in this work. Notice that the latent period is not zero in our simulations. The saccades start at 120 ms. The termination time of the saccades solely depends on the duration of burst under the time-optimal control strategy. The selection of the duration of the burst is in accord with the saccade duration-saccade magnitude characteristic of the main-sequence diagrams [3].

**Figure 10:**
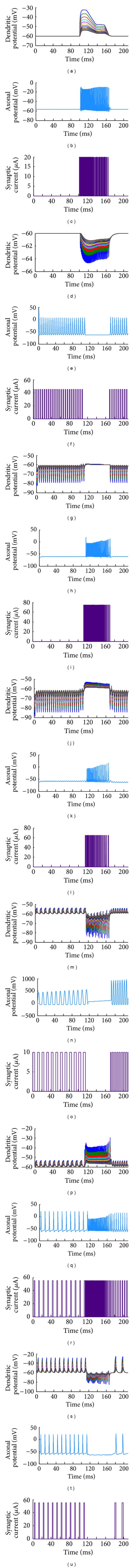
The dendritic membrane potential in mV (first column), axonal membrane potential in mV (second column), and the synaptic pulse current train in *μ*A (third column) of each neuron in a 16° ipsilateral saccade neural controller: (a)–(c) LLBN, (d)–(f) OPN, (g)–(i) EBN, (j)–(l) IBN, (m)–(o) IN, (p)–(r) AN, and (s)–(u) ON. Each neuron fires in harmony with the others in generating this saccade.

For sample illustrations, the plots of dendritic membrane potential (first column), axonal membrane potential (second column), and synaptic current pulse train (third column) of the ipsilateral burst neurons and IN in generation of the 16° saccade are shown in Figure 10. Recall that the train of action potentials is converted to a train of the current pulses in the presynaptic terminal of the neuron to provide excitatory or inhibitory input to the succeeding neurons based on the neural connections in Figure 1. This current pulse flows through the postsynaptic dendritic compartments of the latter neurons, thus providing the smooth postsynaptic potentials to prime the axonal compartment. It appears that upon increasing the stimulus current pulse magnitude, the depolarization of the postsynaptic membrane intensifies.

It is obvious that the synapse propagation raises different excitatory and inhibitory postsynaptic potentials in the dendritic compartments of each postsynaptic neuron (shown in the first column of Figure 10). One can realize that, in view of the trajectory of changes in the membrane potential among the compartments, each postsynaptic neuron, in turn, can either become closer to firing an action potential chain or be inhibited from firing. It is clear that, as the presynaptic input pulses are closely spaced in time, each succeeding postsynaptic potential is smaller than the basic single-pulse response, but the postsynaptic response to each input pulse is demonstrable.

It is worth noting that the LLBN membrane response is different from the rest, since it is stimulated by the contralateral SC current input formerly introduced. Furthermore, the burst onset and offset for each premotor neuron in Figure 10 agreed with its place within saccadic circuitry's hierarchical processing order in generating the final motoneuronal signals. When the ipsilateral EBN is weakly stimulated by the contralateral FN, it renders a special membrane property that tends to a high-frequency burst mechanism until inhibition from the contralateral IBN and the OPN. The EBN synapse consequently provides an excitatory input to the TN and the AN. Recall that the IN is responsible for keeping the agonist and antagonist muscles steady during the periods of fixation. During the pulse phase, however, the ipsilateral TN is inhibited by the contralateral IBN, while the ipsilateral IN is inhibited by the ipsilateral IBN. The IN forms an excitatory synapse with the AN to provide it with the step component of the innervation. Obviously, the burst-tonic firing activity of the AN (Figure 10(q)) reflects the burst firing of the EBN and the tonic firing of the IN.

**Figure 11:**
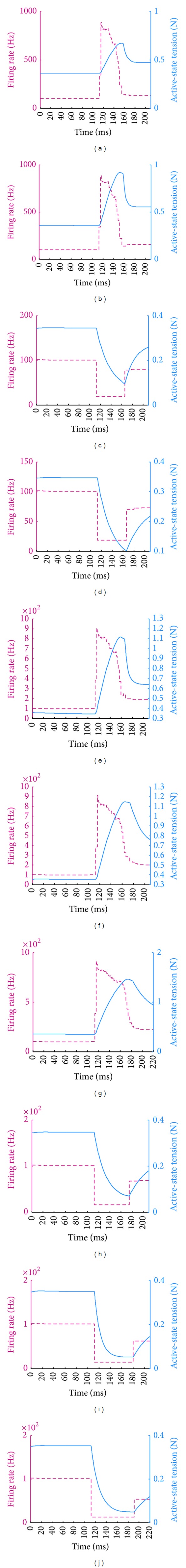
The ipsilateral control simulation results for the agonist and antagonist neural control inputs (dashed) and the corresponding active-state tensions (solid) plotted on the same graph: 4°saccade ((a) and (c)), 8° saccade ((b) and (d)), 12° saccade ((e) and (h)), 16° saccade ((f) and (i)), and 20° saccade ((g) and (j)). The agonist and antagonist controller models provide the active-state tensions to the muscle fiber oculomotor plant.

Presented in Figure 11 are the ipsilateral agonist (first and third rows) and antagonist (second and last rows) burst-tonic firing rates with their respective active-state tensions for the saccades. It is of interest to note that the firing rate of each AN in all scenarios does not vary as a function of saccade magnitude, thus proving that the proposed time-optimal controller is well capable of mimicking the physiological properties of the saccade. The agonist and antagonist active-state tensions during the periods of fixation are found as functions of eye position at steady state (see page 47 in [3]). The corresponding tonic firing rates are readily determined based on a linear transformation that scales the tonic firing rate to the active-state tension [3]. From Figure 11, it follows that the agonist-antagonist firing patterns fairly well match the estimated waveforms based on the system identification approach (see Figure 1.19 in [3]).

**Figure 12:**
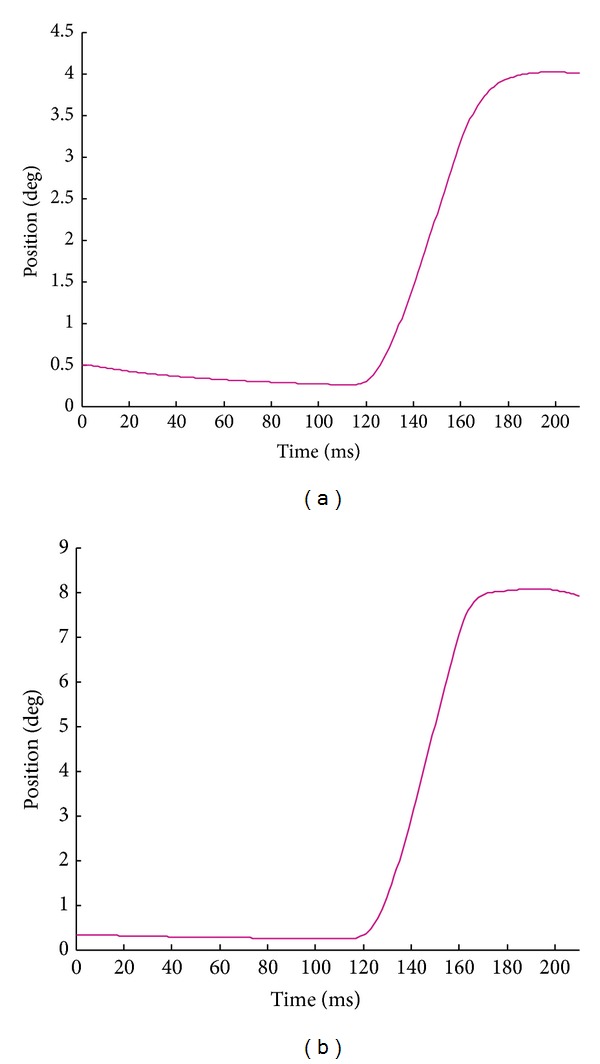
The ipsilateral control simulation results for the monkey small saccades generated by the proposed first-order time-optimal neural saccade controller and the muscle fiber oculomotor model: (a) 4° saccade and (b) 8° saccade. Note that the saccade onset is 120 ms for all cases, but the end time of each saccade differs from the others.

The ipsilateral control simulation results of eye position for the two small saccades under the time-optimal control strategy are demonstrated in Figure 12. The parameterized saccadic oculomotor plant for monkey has been used (see page 47 in [3]). The trend of changes in muscle tensions involved in each saccade is such that neuron-data-derived active-state tensions drive the muscle fiber oculomotor plant.

**Figure 13:**
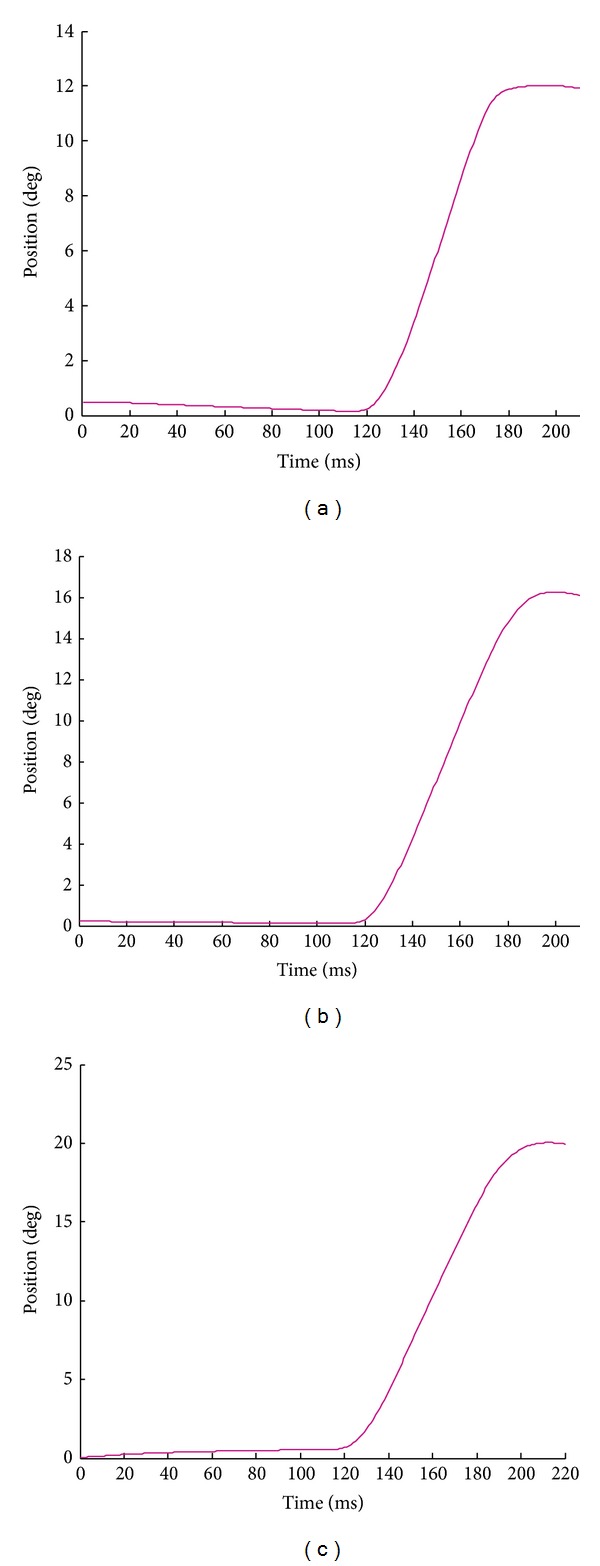
The ipsilateral control simulation results for the monkey large saccades generated by the proposed first-order time-optimal neural saccade controller and the muscle fiber oculomotor model: (a) 12° saccade, (b) 16° saccade, and (c) 20° saccade. Note that the saccade onset is 120 ms for all cases, but the end time of each saccade differs from the others.

Shown in Figure 13 are the ipsilateral control simulation results of eye position for the three large saccades under the time-optimal control strategy. It is of interest to note that, as envisioned [1, 18], the investigated oculomotor plant does not considerably influence the main-sequence diagrams. The entire neural stimulation signals and eye movements on the contralateral side were in close coordination with their corresponding ipsilateral signals for all the simulated conjugate saccades.

## 7. Discussion

The simulation results show remarkable agreement with those provided by analytical descriptions of the agonist and antagonist neural inputs and the corresponding active-state tensions (see Figure 1.19 in [3]). The trajectory of variation in the agonist pulse magnitude among these saccades is consistent with the agonist pulse magnitude-saccade magnitude characteristic for the large saccades (refer to Figure 1.25(A) in [3]). The burst duration is found to show similar correlation with the MLBN duration of burst firing from the extracellular single-unit recordings [24].

As evident by different firing rate trajectories for the EBN, this neuron has tightly coupled characteristics to the saccade [3]. For the saccades examined herein, the initial duration of the EBN firing remained constant among them. However, the duration of the second portion of the burst discharge (gradual drop) varied among them based on the entire duration of the burst firing in Table 3. As indicated in Table 1, the EBN firing lags behind the saccade by 6–8 ms, whereas the AN starts burst firing 5 ms before the saccade (see Figure 10). Finding the dendrite parameters for both of these neurons in meeting the required onset time delay was experimentally challenging.

Implementing the OPN dendrite and synapse models in order that this neuron stops inhibiting the EBN about 10 ms before the saccade and resumes its inhibition almost when the saccade ends was subject to numerous experimental tunings (see Table 1). Without this coordination in timing of the burst firing in the EBN, this neuron can show the rebound burst firing activity. This rebound burst, in turn, causes the saccade to deviate from the normal characteristics. It also was vital that the end of the IBN inhibition of the antagonist motoneurons coincides with the resumption of tonic firing in them such that no deviation from the normal saccade is present.

While the midbrain coordination mechanism in generating saccades is qualitatively studied [13, 15], complete neural circuitry that includes both the premotor and motor neurons in quantifying the final motoneuronal command to eye muscles has not yet been attended. The utility of SNNs to the biophysical modeling of interconnected neurons [4, 5] elucidates broad insights into modeling at higher structural scales, such as the circuit [3, 6, 7] and the systems levels [8]. The computer simulations of neural circuitry herein allow synaptic stimuli to propagate through the saccade pathways so that the motoneurons ultimately drive the oculomotor plant.

A time-optimal neuronal control strategy for human saccadic eye movements was first proposed based on experimental data analysis [25]. We used the first-order time-optimal controller [3] that includes the activation and deactivation time constants in agonist and antagonist controller inputs to the muscle fiber oculomotor plant. This controller has been proven to agree with the experimental findings [25, 26]. Realization of the suitable time constants for both the agonist and antagonist controllers, as expressed in (9) and (11), was key in providing the required steady-state active-state tensions to the muscle fiber oculomotor plant. The estimated activation and deactivation time constants from the system identification approach [1, 3] best satisfy this specification. Without such appropriate parameters, the simulated saccade could be showing deviations from the desired position at steady state.

The set of agonist-antagonist control inputs to the muscle fiber oculomotor plant supports the time-optimal controller in which the motoneurons' firing rate does not determine the saccade magnitude. The application of the MFM in the oculomotor plant proves important in accommodating the constraint on the number of active neurons firing maximally in controlling the saccade magnitude. The number of the active neurons is a key parameter whose adjustment in the MFM is vital in providing the desired saccade control simulation results. It follows from our observations that the duration of the agonist burst firing and the number of active agonist neurons are integral to determining the saccade size (see Table 3).

It is noteworthy that the duration of agonist burst discharge is of prime significance in determining the saccade magnitude as seen in Figure 11. It is concluded that the neural network is constrained by a minimum duration of the agonist pulse and that the most dominant factor in determination of the amplitude is the number of active neurons for the small saccades. For the large saccades, however, the duration of agonist burst firing is directly related to the saccade magnitude. The number of active neurons for the 16° and 20° saccades remains relatively the same, although the 12° saccade aggregates fewer active neurons as seen in Table 3. The discussion by Enderle and Sierra [12] is enlightening as to the increasing movement field of activity within the SC for saccades up to 12° for the monkey data. Furthermore, from the velocity profiles for the simulated saccades, it was found that monkey saccade has larger peak velocity than that of the human [12].

The final eye position results establish evidence for the acceptable performance of the proposed neural circuitry and the exploited time-optimal controller in modeling the horizontal monkey saccades. The dependence of these different saccades on the agonist pulse duration has been found to be well presented by our time-optimal controller. The simulation results substantiate the time-optimal controller by the close agreement obtained with the analytical solutions of saccade characteristics [3, 12]. This agreement gives rise to the accuracy of the experimentally found membrane parameters in modeling of each neuron listed in Table 2.

## 8. Conclusion

We simulated five different conjugate goal-directed horizontal monkey saccades: 4°, 8°, 12°, 16°, and 20°, under the first-order time-optimal control strategy. A parallel-distributed neural network model of the midbrain was first presented. To develop the quantitative computational models that establish the basis of this functional neural network model, we next described the saccade burst generator dynamics. A neural circuit model was then demonstrated and parameterized to match the firing characteristics of eight neuron populations at both the premotor and motor stages. In this context, we elevated the neural modeling from a single neuron to a network of neurons. Our control strategy was to define two controllers, namely, agonist and antagonist controller models, characterized by the pulse-slide-step and pause-step waveforms, respectively. The horizontal monkey saccades were well characterized by integrating the neural controllers into a third-order linear muscle fiber oculomotor plant. 100 identical muscle fibers were connected in series in both the agonist and antagonist muscles in the oculomotor plant. Under the time-optimal strategy, the number of neurons that actively fire and the duration of the agonist pulse determined the saccade magnitude. The choice of the number of active neurons proved accurate in adapting the muscle fiber model to provide the desired control simulation results. The proposed saccadic circuitry is thus a complete model of saccade generation since it not only includes the neural circuits at both the premotor and motor stages of the saccade generator, but also uses a time-optimal controller to yield the desired saccade magnitude for both the small and large saccades. The saccade characteristics were found to be well correlated with those found by analytical descriptions and experimental data.

## Figures and Tables

**Table 1:** Firing activity of neural sites during an ipsilateral saccade [3].

Neural site	Burst onset before saccade (ms)	Peak firing rate (Hz)	Burst end with respect to saccade end
Contralateral SC	20–25	800–1000	Almost the same
Ipsilateral LLBN	20	800–1000	Almost the same
OPN	6–10	150–200 (before and after)	Almost the same
Ipsilateral EBN	6–8	600–1000	∼10 ms before
Ipsilateral IBN	6–8	600–800	∼10 ms before
Ipsilateral TN/IN	5	Tonic firing (before and after)	It resumes tonic firing when saccade ends
Ipsilateral AN	5	400–800	∼5 ms before
Ipsilateral FN	20	Pause during saccade and a burst of 200 Hz near the end of the saccade	Pause ends with burst ∼10 ms before saccade ends; it resumes tonic firing ∼10 ms after saccade ends
Contralateral FN	20	200	Pulse ends with pause ∼10 ms before saccade ends; it resumes tonic firing ∼10 ms after saccade ends
Ipsilateral cerebellar vermis	20–25	600–800	∼25 ms before
Ipsilateral nucleus reticularis tegmenti pontis	20–25	800–1000	Almost the same
Ipsilateral substantia nigra	40	40–100	It resumes firing ∼40–150 ms after saccade ends

**Table 2:** Parametric realization of eight distinct neurons in terms of dendritic, axonal, and synaptic behaviors.

Neuron	Dendrite		Axon		Synapse
	Capacitor (*μ*F)	Resistor (kΩ)	Firing threshold voltage (mV)	Coefficient	Pulse amplitude (µA)
LLBN	0.5	3.75	−45	18,000	20
OPN	1.0	6.3	−60	1,800	45
EBN	0.45	3.1	−60	35,000	75
IBN	0.35	4.5	−45	15,000	65
AN	0.35	5.5	−45	17,000	55
ON	0.45	4.0	−45	17,000	55
TN	0.35	4.5	NA	NA	10
IN	0.4	4.5	NA	NA	10

**Table 3:** Controlling the saccade magnitude with the duration of burst firing and the number of active neurons.

Saccade magnitude (degrees)	Burst duration (ms)	Number of active neurons
4	40	48
8	42	76
12	52	75
16	56	100
20	65	92
